# Comparison of the McGRATH^TM^ Video Laryngoscope and Macintosh Laryngoscope for Orotracheal Intubation in a Simulated Difficult Airway Scenario: An Open-Label, Randomized Clinical Trial

**DOI:** 10.3390/medicina59020282

**Published:** 2023-01-31

**Authors:** Jong-Yeop Kim, Seonghyeok Park, Minho Oh, Jong-Bun Choi, Hyun-Ji John, Soo-Kyung Lee, Yi-Hwa Choi

**Affiliations:** 1 Department of Anesthesiology and Pain Medicine, School of Medicine, Ajou University School of Medicine, Suwon 16499, Republic of Korea; 2 Department of Anesthesiology and Pain Medicine, School of Medicine, Hallym University Sacred Heart Hospital, Anyang 14068, Republic of Korea

**Keywords:** airway management, anesthesia, general, laryngoscopes, intubation

## Abstract

*Background and Objectives*: Difficult intubation, which may be encountered unexpectedly during anesthesia, can increase patients’ morbidity and mortality. The McGRATH video laryngoscope is known to provide improved laryngeal visibility in patients with difficult or normal airways. The purpose of this study was to evaluate the efficacy of the McGRATH video laryngoscope for orotracheal intubation compared with that of conventional Macintosh laryngoscopes in simulated difficult airway scenarios. *Materials and Methods*: In this randomized controlled trial, patients who were scheduled for surgery under general anesthesia requiring orotracheal intubation were assigned to the Macintosh laryngoscope (n = 50) or McGRATH video laryngoscope (n = 45) groups. In this study, to create a simulated difficult airway condition, the subjects performed manual in-line stabilization and applied a soft cervical collar. The primary outcome was the rate of successful intubation within 30 s. The time required for an intubation, glottis grade, intubation difficulty scale (IDS score), the subjective ease of intubation, and optimal external laryngeal manipulation (OLEM) were evaluated. In addition, complications caused by each blade were investigated. *Results*: The intubation success rate within 30 s was not significantly different between the two groups (44 (88.0%) vs. 36 (80.0%), *p* = 0.286). The glottic grade was better in the McGRATH group than in the Macintosh group (*p* = 0.029), but neither the intubation time (26.3 ± 8.2 s vs. 24.2 ± 5.0 s, *p* = 0.134) nor the rates of oral bleeding (2 (4.0%) vs. 0 (0.0%)) and tooth injury (0 (0.0%) vs. 1 (2.2%)) were significantly different between the two groups. *Conclusions*: The use of the McGRATH video laryngoscope did not improve the intubation success rate or shorten the intubation time. However, the McGRATH video laryngoscope provided a better glottis view than the conventional Macintosh laryngoscope in patients with a simulated difficult airway.

## 1. Introduction

Difficult intubation, which may be encountered unexpectedly under general anesthesia, is a life-threatening event and increases the morbidity and mortality of anaesthetized patients during surgery [1]. Recently, a number of devices have been used in the anesthetics field to cope with unanticipated difficult airways to ensure the safety of patients during intubation. Video laryngoscopes are known to provide a better visualization of laryngeal structures than conventional laryngoscopes [2,3,4] and are used as an alternative option for the management of difficult airways.

The McGRATH video laryngoscope (Aircraft Medical Ltd., Edinburgh, UK) is a portable video laryngoscope with a liquid crystal display (LCD) monitor and a disposable curved blade. Among video laryngoscopes, the McGRATH video laryngoscope is known to provide excellent laryngeal visibility in cases of anticipated-difficult and anticipated-unsuccessful tracheal intubation as well as during normal airway management [5,6]. In addition, the McGRATH video laryngoscope is widely used for educational purposes, because it allows supervision of the trainee through a video monitor and it enables feedback to be given to them promptly. These devices have also been used increasingly in routine intubation procedures in several institutions during the recent pandemic to lower the risk of infection through multiple channels associated with intubation. However, there is a paucity of prospective studies on unexpected difficult airways under general anesthesia, except for a study of a normal airway, or in a manikin for training models, using the McGRATH video laryngoscope. Due to the nature of difficult airway management, the patient may be at risk within a short time. For this reason, it is difficult to prospectively investigate difficult intubation in actual clinical settings.

To the best of our knowledge, compared to that of direct laryngoscopy, the success rate of intubation and the time required for predicted difficult intubation have reported conflicting results in previous studies [7,8,9]. In this study, to create a simulated difficult airway condition, the subjects performed manual in-line stabilization [8] and applied a cervical collar during endotracheal intubation. Thus, the purpose of this study was to evaluate the efficacy of the McGRATH video laryngoscope for orotracheal intubation in comparison to that of conventional Macintosh laryngoscopes in a simulated difficult airway scenario, with manual in-line stabilization and application of a semi-rigid cervical collar. We aimed to investigate the intubation success rate, time to intubation, glottis grade, and self-reported ease of intubation in both groups during endotracheal intubation under general anesthesia.

## 2. Materials and Methods

This study was a prospective, randomized, patient-blinded trial from July 2017 to August 2018 after obtaining approval from the Institutional Review Board of the Hallym University Sacred Heart Hospital (IORG0004993, IRB00005964, approved date: 25 April 2017), and it was internationally registered for clinical trials (ClinicalTrials.gov unique identifier: NCT03516539). Written informed consent for this study was obtained from all participants before the day of a scheduled surgery.

We initially screened 100 patients aged 19 to 70 years, using the American Society of Anesthesiologists physical status classification system (ASA) 1 or 2, who were scheduled for surgery requiring orotracheal intubation under general anesthesia at our institution. The exclusion criteria were as follows: a requirement for rapid sequence intubation, cervical instability or cervical spine injury, morbid obesity (body mass index > 40 kg/m^2^), risk of pulmonary aspiration, increased bleeding tendency, and difficulties in communication. Baseline characteristic data of all study patients were collected before the scheduled surgery. Patient characteristics, including age, sex, weight, height, and body mass index (BMI), were recorded. The airway of each patient was assessed, including Mallampati score, thyromental distance, maximal mouth opening (inter-incisor distance), cervical spine mobility (normal/reduced/fixed flexion), and the status of upper incisors (absent/normal/prominent), preoperatively. Patients were randomly allocated to the Macintosh group (Group DL) or the McGRATH group (Group ML) using a computer-generated random numbers table. The randomization sequence was generated with a block size of 4. 

All patients were premedicated with glycopyrrolate 0.1 mg intramuscularly just before entering the operating room. All patients were monitored in the operating room through electrocardiography, non-invasive arterial blood pressure measurement, pulse oximetry, capnography, and a Bispectral Index^®^ monitor (A-3000 EEG BIS monitor, Aspect Medical Systems, Norwood, MA, USA) on the forehead of each patient. Thereafter, baseline hemodynamic data were obtained.

After pre-oxygenation with 100% oxygen for 2 min, propofol (effect site concentration, 5.0 μg/mL) and remifentanil (effect site concentration, 4.0 μg/mL) were administered through a target-controlled infusion (TCI) pump (Orchestra^®^ target-controlled infusion pump, Fresenius Vial, Brezins, France). When the patients did not respond to verbal stimulus, rocuronium (0.6 mg/kg) was injected intravenously, and manual mask ventilation was conducted with 100% oxygen for 2 min. After a train-of-four of zero and a BIS value < 60, transoral endotracheal intubation was performed using a McGRATH video laryngoscope (Aircraft Medical Ltd., Edinburgh, UK) or direct conventional Macintosh laryngoscope (Welch Allyn fiber optic laryngoscope handle (60813) and blades 3,4; New York, NY, USA) according to the random table. The patient was laid supine with nothing under the patient’s head and manually stabilized in-line by an anesthetist using a semi-rigid cervical collar in both groups to limit movement of head and cervical spine during intubation. In both groups, a blade size of 4 was used for the male patients, and a blade size of 3 was used for the female patients. At this time, after preloading the intubating stylet (Shiley^TM^ intubating stylet, Mansfield, MA, USA) with slightly bent distal tip, standard Mallinckrodt^®^ tracheal tubes (Shiley^TM^, TaperGuard Oral/Nasal tracheal tube, Covidien, Mansfield, MA, USA) were used with sizes 7.0 mm and 7.5 mm for females and males, respectively, as is routine practice at our institution. When the endotracheal tube had passed through the vocal cords, the stylet in the tube was immediately removed by a trained nurse. The patients were intubated by one anesthesiologist with over 10 years of anesthetic experience and proficiency in video laryngoscopy, while the time required for intubation was recorded by the other medical personnel who were not engaged in this study. The intubation time was measured from the passage of the laryngoscopy tip past the patient’s incisors until the appearance of an end tidal CO_2_ trace on the capnography monitor for confirmation of successful intubation. After intubation, the glottis grade, Cormack–Lehane laryngeal visual field, external laryngeal manipulation, intubation difficulty scale (IDS) [10], and a subjective report of intubation difficulty (easy/moderate/difficult) were recorded. Intubation difficulty was based on the response of an experienced anesthesiologist who performed the intubation. Complications from the use of each blade were also evaluated by a blinded anesthetist, based on the presence of any blood on the laryngoscope blade or perioral soft tissues, oropharyngeal bleeding, tooth or lip injury. The grade of oropharyngeal bleeding was classified as none, trace, moderate, or severe. Hemodynamic profiles, including mean arterial pressure, heart rate, arterial oxyhemoglobin saturation, and bispectral index (BIS) values were recorded immediately after entering the operating room, 1 min after induction, just before intubation, and 1 min after intubation.

The primary outcome variable was the intubation success rate. Successful intubation was defined as tracheal intubation within 30 s at the first attempt [10,11]. The secondary outcomes were successful intubation within <60 s, the time to intubation, the glottis grade, and the ease of intubation during orotracheal intubation under general anesthesia with a McGRATH video laryngoscope and direct Macintosh laryngoscope.

Categorical variables are shown as frequencies with percentages, and continuous variables are shown as medians and interquartile ranges or means and standard deviations. The categorical variables were compared using the chi-square test, and the continuous variables were analyzed using Student’s *t*-test. Based on previous studies, we assumed that the first attempt success rate of the Macintosh laryngoscope in cases of difficult airways would be 59% [8,12], and it would be improved to 90% using the McGRATH video laryngoscope [13,14]. It was estimated that 50 patients in each group would be needed for a probability of alpha error of 5%, beta error of 10% and considering the possibility of participants drop-out rate of 10%. All statistical analyses were performed using SPSS (version 21.0, IBM, Chicago, IL, USA), and statistical significance was set at *p* < 0.05.

## 3. Results

**Figure 1:**
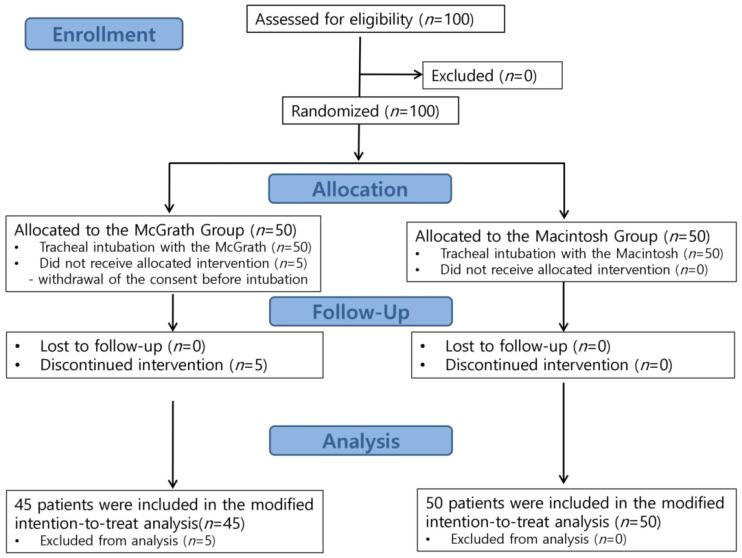
Study flow diagram.

The overall study progress is shown in Figure 1 as a flow diagram including screening, randomization, and assignment to either group. During the study period, we initially screened 100 patients. A total of 50 patients were randomly assigned to the McGRATH group, and 50 patients were assigned to the Macintosh group. Five patients (three male and two female patients) in the McGRATH group withdrew consent immediately after randomization. Therefore, 95 patients (45 in the McGRATH group and 50 in the Macintosh group) were finally included in the modified intention-to-treat analysis.

The baseline characteristics of the patients are summarized in Table 1. The age (50.2 ± 13.2 vs. 49.3 ± 15.2 years, *p* = 0.750) and sex distribution (64.0% vs. 46.7% males, *p* = 0.136) were not significantly different. The airway assessment measures associated with difficult intubation, such as Mallampati grade (*p* = 0.437), thyromental distance (8.6 ± 1.4 vs. 8.6 ± 1.3, *p* = 0.952), mouth opening (3.7 ± 0.6 vs. 3.6 ± 0.7, *p* = 0.721), neck mobility (*p* = 0.522), and upper incisor status (*p* = 0.452), were not statistically different. The primary analysis of each device is presented in Table 2. In the analysis of the primary study outcome (the rate of successful intubation within 30 s), there was no significant difference between the two groups (44 (88.0%) vs. 36 (80.0%), *p* = 0.286). Successful intubation within 60 s was also not significantly different (44 (97.8%) vs. 47 (94.0%), *p* = 0.686). In the Macintosh group, three patients met the criteria for intubation failure (more than 60 s to intubate). Two patients were intubated with the assigned Macintosh laryngoscope after removal of their cervical collars. One patient was successfully treated with a Schucman blade (Schucman-Pro size 3.5, Truphatek^®^, Ambala, India). In the McGRATH group, one patient took more than 60 s to be intubated. The patient was intubated after removing the cervical collar and applying optimal external laryngeal manipulation (OLEM) using the assigned McGRATH laryngoscopy. The overall intubation time (24.2 ± 5.0 vs. 26.3 ± 8.2 min, *p* = 0.134) was slightly shorter in the McGRATH group than in the Macintosh group, without statistical significance. The perceived intubation difficulty was not significantly different between the two groups. Only one patient had more than one attempt in the McGRATH group, and three patients had more than one attempt in the Macintosh group. All patients were in the failed intubation group. The IDS score (median 0 (IQR, 0–1.0) vs. 1.0 (IQR, 1.0–3.0), *p* = 0.003) and glottis grade (*p* = 0.029) were lower in the McGRATH group than in the Macintosh group. No major complications occurred. The rates of oral bleeding (2 (4.0%) vs. 0 (0.0%)) and tooth injuries (0 (0.0%) vs. 1 (2.2%)) were also not significantly different.

During all periods prior to tracheal intubation, hemodynamic parameters, including mean arterial pressure (MAP), heart rate (HR), oxygen saturation (SpO_2_), and bispectral index (BIS), were not significantly different between the two groups. During the post-intubation period, the changes in MAP (27.0 ± 23.7 vs. 26.1 ± 24.2, *p* = 0.851), HR (15.4 ± 14.7 vs. 17.3 ± 15.4, *p* = 0.517), and SpO_2_ (−0.2 ± 1.7 vs. −0.3 ± 0.6, *p* = 0.473) were not significantly different between the two groups. The change in sedation depth after intubation (4.7 ± 9.7 vs. 4.5 ± 11.3, *p* = 0.917) was also not significantly different between the two groups.

## 4. Discussion

In this single-center randomized controlled comparative study, the McGRATH video laryngoscope showed similar efficacy in terms of the first attempt success rate within 30 s and intubation time as compared with the conventional Macintosh laryngoscope for orotracheal intubation in patients with in-line manual stabilization using a neck collar. Meanwhile, the McGRATH group showed better glottis visualization and lower IDS scores than that of the Macintosh group in this study.

Previous studies of simulated difficult airway conditions have shown inconsistent results regarding the success rate and intubation time between the McGRATH video laryngoscope and the Macintosh laryngoscope [7,8,9]. Foulds et al. reported that the McGRATH video laryngoscope had a statistically significant lower intubation failure rate (successful intubation: McGRATH group 24 (100%) vs. Macintosh group 18 (72%), *p* = 0.017) and no difference in intubation time (time to intubate: McGRATH group 45 s (30–95) vs. Macintosh group 60 s (37.8–56.3), *p* = 0.125), than the Macintosh laryngoscope in patients with cervical spine immobilization using a semi-rigid cervical collar [7]. In another study, the McGRATH video laryngoscope had an increased intubation success rate (McGRATH group 44 (100%) s vs. Macintosh group 26 (59%), *p* < 0.001), and prolonged intubation time (McGRATH group 35.8 ± 20.4 s vs. Macintosh group 21.7 ± 9.4 s, *p* < 0.0001), compared with that of the Macintosh laryngoscope in manual in-line stabilization [8]. In contrast, Ilyas et al. found that McGRATH video laryngoscopy led to more intubation failure (McGRATH group 5 vs. Macintosh group 0) and prolonged intubation time (McGRATH group 82.7 s (80.0) vs. Macintosh group 50.0 (32.6), *p* < 0.0003) compared with that of the Macintosh laryngoscope [9]. In our study, we used a stringent cut-off time of 30 s to verify the utility of McGRATH video laryngoscope more clearly [10,11]. Although numerically higher (88% vs. 80%), the statistically significant benefits of the McGRATH video laryngoscope were not shown because of an unexpectedly higher success rate in the control group. The overall result of our study was similar to that of a multicenter study of 720 patients, in which the McGRATH video laryngoscope showed a 98% success rate within 60 s on the first attempt (117 out of 120, *p* < 0.05) in a simulated difficult airway [15]. A recent study reported that the McGRATH video laryngoscope provided a similar success rate (risk ratio, 1.00; confidence interval (CI), 0.95–1.05) but extended time to intubation (mean difference, 10.1 s; CI, 2.74–17.5) as the Macintosh laryngoscope [16].

The McGRATH video laryngoscope is associated with technical issues, such as non-intuitive hand–eye coordination through an indirect liquid crystal display (LCD) view, unlike the Macintosh direct laryngoscope, despite a better glottic view [16]. The blade of the McGRATH video laryngoscope is more acutely angulated than the conventional laryngoscope. At this point, it is known that the tip of the endotracheal tube is located posteriorly to the glottis during McGRATH video-laryngoscopy-guided intubation. Therefore, the screened view of the video laryngoscope is mismatched with the direct view of a conventional laryngoscope and the axis of oral-laryngeal-pharyngeal anatomy is unfamiliar [17]. All subjects in this study were intubated with a pre-loaded stylet. During the study, one anesthetist (Y.H.C.), with over 10 years’ experience, conducted all tracheal intubations to eliminate technical differences. However, in over 25 cases, the operator became more proficient in McGRATH video laryngoscopy, and the time for intubation was less than that in the earlier period of the study. In this study, the overall intubation time (24.2 ± 5.0 vs. 26.3 ± 8.2 min, *p* = 0.134) was not significantly different between the two devices. This result is similar to the reported median time of 24.7 s for an intubation by experienced anesthetists using the McGRATH video laryngoscope [6].

The McGRATH video laryngoscope provided a better view of the glottis than the conventional laryngoscope in this study, which is consistent with previous reports [18,19,20,21]. Su et al. reported that the view of glottis was significantly improved using the video laryngoscope compared with direct laryngoscopy [18]. In another study, the incidence of a grade 1 glottic view was higher in the McGRATH group (McGRATH group 83% vs. Macintosh group 57%, *p* = 0.019) in patients with an expected normal airway [19]. In a study of 120 patients for nasotracheal intubation, it was also found that the percentage of the glottic opening scores in the McGRATH video laryngoscopy were significantly superior to those in the Macintosh group (Cormack–Lehane (CL) grade 1; 21/40 (52.5%) vs. 1/40 (2.5%)) [20]. However, the superiority of the first attempt intubation success rate in difficult airway conditions has not been proven in this study. In general, when poor visualization of the glottis is encountered, an anesthetist considers that it would be difficult to intubate. However, it should be noted that a better glottis view does not always guarantee a successful intubation, especially in a video laryngoscopy. Adnet et al. reported that poor visualization of the glottis alone is not a determining factor for difficult intubation. In their study, 21/34 (61.7%) intubations with a Cormack grade > or = to III were completed on the first attempt. Four intubations with Cormack grade I glottic visualization were judged very difficult, subjectively and by IDS [22]. Additionally, Gu et al. reported that a good glottis view made intubation more difficult on a hyperangulated GlideScope. Hyper-curved or angulated blades with video laryngoscopy can be effective for obtaining a view of the larynx in cases of difficult airways. Unfortunately, the maximizing glottic exposure by GlideScope^®^ video laryngoscope makes tracheal intubation more difficult. In their study, a deliberately restricted view of the larynx allowed a significantly faster time to intubation compared to the conventional full view of the larynx using the GlideScope^®^ video laryngoscope, due to the dropping of the glottis when the viewing angle is reduced [23]. Additionally, Niforopoulou et al. reported that, despite the very good visualization of the glottis structure, the insertion and advancement of the endotracheal tube using video laryngoscopy can occasionally fail [17].

The McGRATH video laryngoscope requires a lesser lifting force in the same glottis grade as compared to conventional laryngoscopes during this study. Five patients in the Macintosh group required more lifting force during intubation as compared to the other patients, whereas none of the patients in the McGrath group required additional lifting force. The lifting force could increase the risk of a laryngoscopy-associated injury to the soft palate or oropharynx. The peak lifting force on the base of tongue induces stress responses which may be harmful to susceptible patients. Increased lifting force was reported in GlideScope^®^ video laryngoscope [24]. However, the lifting forces (peak, average and impulse) on the base of tongue during laryngoscopy and tracheal tube delivery were less with the GlideScope^®^ video laryngoscope compared with the Macintosh laryngoscope. Unfortunately, we did not record the lifting force with an objective index using a force measurement, but still reported that the McGRATH video laryngoscope required less lifting force than the conventional Macintosh video laryngoscope compared to the study outcome of the GlideScope^®^ video laryngoscope. The position of the head and neck is important for optimizing the laryngeal view for tracheal intubation. The modified Jackson position has been recommended as the optimal position for intubation. In this position, the neck is flexed on the chest using a 10–15 cm pillow placed under the nape, and the head is tilted so that the mouth can be opened (sniffing position). In particular, in cases of short-necked and obese patients and torticollis, the position is known to make intubation much easier. In contrast, in order to simulate a difficult airway condition for this study, a semi-rigid cervical collar was used to fix the patient’s cervical spine and conduct manual in-line stabilization without any pillow.

This study has several limitations. First, one anaesthetist conducted all procedures to eliminate technical differences. Because of this, the investigator could not be blinded, leading to a potential bias. In addition, all endotracheal intubations were conducted alone by one skilled anesthetist in this study. In this regard, the investigator’s familiarity with the McGRATH video laryngoscope cannot represent all anesthetist. Second, we hypothesized that the first attempt success rate was 59% in the Macintosh group based on a previous study [8,12]. However, in this study, given that the success rate of 94% in the Macintosh group was significantly higher than the expected rate of 59%, the sample size of 50 patients in each group might not be enough to provide a statistical difference between the Macintosh and McGRATH groups. A study with a larger sample size might be needed for generalized results with greater precision and power. Third, most patients scored 0 for laryngeal pressure, which is one parameter of IDS scoring. This is because the cervical collar was applied to all patients according to the design of this study, and laryngeal pressures cannot be applied, except in failed intubation patients. Thus, the IDS score would be lower than the real value, and underestimation of IDS in this study may have led to a potential bias. A further prospective study is warranted to evaluate the efficacy of the McGRATH video laryngoscope over conventional laryngoscopes in difficult airway conditions in the actual clinical setting.

## 5. Conclusions

In this study, the use of the McGRATH video laryngoscope did not improve the first attempt intubation success rate nor shorten the intubation time in the simulated difficult airway. However, the McGRATH video laryngoscope provided a better glottis view than that of the conventional Macintosh laryngoscope. These findings suggest that the McGRATH video laryngoscope may not guarantee successful intubation in difficult airway conditions despite a better glottis view.

## Figures and Tables

**Table 1:** Patient characteristics.

	Macintosh Group (*n* = 50)	McGRATH Group (*n* = 45)
Age, years	50.2 ± 13.2	49.3 ± 15.2
Male, n (%)	32 (64.0)	21 (46.7)
Body mass index	24.0 ± 3.8	25.0 ± 4.1
Mallampati score		
1	15 (30.0)	13 (28.9)
2	14 (28.0)	18 (40.0)
3	19 (38.0)	11 (24.4)
4	2 (4.0)	3 (6.7)
Thyromental distance(cm)	8.6 ± 1.4	8.6 ± 1.3
Mouth opening (cm)	3.7 ± 0.6	3.6 ± 0.7
Neck mobility		
Normal	48 (96.0)	45 (100.0)
Reduced	2 (4.0)	0 (0.0)
Upper incisor		
Normal	46 (92.0)	42 (93.3)
Absent	4 (8.0)	2 (4.4)
Prominent	0 (0.0)	1 (2.2)

Values are presented as mean ± standard deviation (SD) or number (proportion, %).

**Table 2:** Intubation profiles.

	Macintosh Group (*n* = 50)	McGRATH Group (*n* = 45)	*p*-Value
Intubation success < 30 s	36 (80.0)	44 (88.0)	0.286
Intubation success < 60 s	47 (94.0)	44 (97.8)	0.686
Intubation time (s)	26.3 ± 8.2	24.2 ± 5.0	0.134
Intubation difficulty			0.137
1	33 (66.0)	24 (53.3)	
2	15 (30.0)	14 (31.1)	
3	2 (4.0)	7 (15.6)	
IDS score (IQR)	1.0 (0–3.0)	0 (0–1.0)	0.003 *
Glottic grade			0.029 *
1	16 (35.6)	25 (50.0)	
2	11 (24.4)	18 (36.0)	
3	12 (26.7)	6 (12.0)	
4	6 (13.3)	1 (2.0)	
OLEM	0 (0.0)	1 (2.2)	0.958
Oral bleeding (−/+)	2 (4.0)	0 (0.0)	0.522
Tooth injury (−/+)	0 (0.0)	1 (2.2)	0.958

* *p* < 0.05. Values are presented as mean ± standard deviation (SD), median (interquartile range), or number (%). IDS, intubation difficulty scale; OLEM, optimal external laryngeal manipulation; IQR, interquartile range.

## Data Availability

The data that support the findings of this study are available from the corresponding author upon reasonable request.
